# Integrating ATAC-seq and RNA-seq Reveals the Dynamics of Chromatin Accessibility and Gene Expression in Apple Response to Drought

**DOI:** 10.3390/ijms231911191

**Published:** 2022-09-23

**Authors:** Shicong Wang, Jieqiang He, Mengting Deng, Caixia Wang, Ruifeng Wang, Jinjiao Yan, Minrong Luo, Fengwang Ma, Qingmei Guan, Jidi Xu

**Affiliations:** 1 State Key Laboratory of Crop Stress Biology for Arid Areas/Shaanxi Key Laboratory of Apple, College of Horticulture, Northwest Agricultural and Forestry University, Yangling, Xianyang 712100, China; 2 College of Forestry, Northwest Agricultural and Forestry University, Yangling, Xianyang 712100, China

**Keywords:** apple, drought, chromatin accessibility, gene expression, ATAC-seq, RNA-seq

## Abstract

Drought resistance in plants is influenced by multiple signaling pathways that involve various transcription factors, many target genes, and multiple types of epigenetic modifications. Studies on epigenetic modifications of drought focus on DNA methylation and histone modifications, with fewer on chromatin remodeling. Changes in chromatin accessibility can play an important role in abiotic stress in plants by affecting RNA polymerase binding and various regulatory factors. However, the changes in chromatin accessibility during drought in apples are not well understood. In this study, the landscape of chromatin accessibility associated with the gene expression of apple (GL3) under drought conditions was analyzed by Assay for Transposase Accessible Chromatin with high-throughput sequencing (ATAC-seq) and RNA-seq. Differential analysis between drought treatment and control identified 23,466 peaks of upregulated chromatin accessibility and 2447 peaks of downregulated accessibility. The drought-induced chromatin accessibility changed genes were mainly enriched in metabolism, stimulus, and binding pathways. By combining results from differential analysis of RNA-seq and ATAC-seq, we identified 240 genes with higher chromatin accessibility and increased gene expression under drought conditions that may play important functions in the drought response process. Among them, a total of nine transcription factor genes were identified, including ATHB7, HAT5, and WRKY26. These transcription factor genes are differentially expressed with different chromatin accessibility motif binding loci that may participate in apple response to drought by regulating downstream genes. Our study provides a reference for chromatin accessibility under drought stress in apples and the results will facilitate subsequent studies on chromatin remodelers and transcription factors.

## 1. Introduction

As sessile organisms, plants cannot escape drought but must respond through various signaling pathways. Drought induces dynamic changes in gene expression, followed by the regulation of growth and metabolism. Plant responses to drought include changes in transcriptional regulation, translation, and post-translational regulation. These changes can be mediated by the approaches of alternative splicing, ABA-dependent versus ABA-independent responses, protein phosphorylation, ROS signaling, and epigenetic regulation. Many genes have been reported in these drought response pathways [1,2]. Recent studies have demonstrated the crucial role of epigenetics in plant response to drought. Epigenetic mechanisms include DNA methylation, histone modifications, non-coding RNAs, and chromatin remodeling. DNA assembles with histones into chromatin higher-level structures. Dynamics of chromosome accessibility affect the ability of RNA polymerases and regulatory proteins to bind the DNA, which can alter the transcriptional regulation of stress-related genes and thus the response to drought [3,4]. Hence, the study of the dynamics of chromatin accessibility may help decipher changes in the epigenetic code under drought.

Various transcription factors (TFs) have regulatory roles in drought, such as basic leucine zipper (bZIP), dehydration responsive element binding (DREB), DNA binding with one finger (DOF), heat shock factor (HSF), MYB, NAC, TCP, and WRKY [5]. For example, DREB transcription factors have essential functions in drought-related gene regulation and include the AP2/EREBP or AP2/ERF (APETALA2/ethylene-responsive element-binding protein/factor) TF families [6]. WRKYs are positive regulators of ABA-mediated stomatal closure and regulators of drought response, which are closely related to the ABA pathway [7]. GATA transcription factors are also reported to function in ABA-dependent and independent signaling pathways [8]. MYB3R was reported to be involved in drought-related processes [9]. The binding of transcription factors may be influenced by open chromatin, thus modulating transcriptional regulation of downstream genes. The study of open chromatin under drought is important to reveal the regulatory role of transcription factors.

For chromatin accessibility studies, drought-induced changes in chromatin have focused on chromatin remodeling enzymes. Chromatin remodeling ATPases use energy generated by ATP hydrolysis to alter histone-DNA interactions. Among four well-characterized subfamilies of ATP-dependent chromatin remodeling enzymes, only SWI/SNF and CHD have been found to be involved in the water stress response of plants [3]. Three types of SWI/SNF subfamily chromatin remodeling ATPases are found in plants, BRAHMA (BRM), SPLAYED (SYD), and MINUSCULE (MINU) [3]. BRM can bind to the promoter of *ABI5* by affecting the stability of the nucleosome [10]. The SWI2/SNF2 ATPase component BRM is a critical target for the ABA-dependent dephosphorylation switch that is manipulated by essential genes *PP2CA* and *SnRK2* of the ABA pathway [11]. Mutants of *SWI3B* have reduced ABA sensitivity and reduced expression of *RD29B* and *Rab18*. SWI3B interacts with HAB1, ABI1, ABI2, and PP2C, which act as negative regulators of ABA signaling [12]. MINU1/AtCHR12 ATPase is related to growth under stress in Arabidopsis, and overexpression of *MINU1* under drought conditions leads to growth arrest [13]. Mutants of LIKE HETEROCHROMATIN PROTEIN 1 (*LHP1*), a reader protein of H3K27me3, increased ABA sensitivity and drought resistance [14]. PICKLE (PKL) is the most well-characterized CHD chromatin-remodeling enzyme in Arabidopsis. The expression of *ABI3* and *ABI5* in *PKL* mutants under ABA treatment decreased the occupancy of H3K9me2 and H3K27me2 on their promoters [3,15]. However, how to reveal changes in chromatin accessibility at the genomic level is important for the study of chromatin accessibility.

With advances in high-throughput sequencing technology, various methods have been developed to study chromatin accessibility. ATAC-seq is a popular method for determining chromatin accessibility across the genome. This approach has high sensitivity and specificity and requires few cells. ATAC-seq has been widely applied in many plants to predict cis-acting elements and chromatin accessibility [16,17,18]. Additionally, ATAC-seq has been used to assess changes in chromatin accessibility during stress responses in different plants. For example, ATAC-seq and RNA-seq were used to identify nine cold-responsive transcription factors, including CBF4, RAV1, and ERF104 in grapes with changes in chromatin accessibility and gene expression [19]. A comprehensive profile of nuclear dynamics in rice under heat stress was revealed by a co-analysis of ATAC-seq and RNA-seq, providing insight into changes in chromatin structure and transcriptional regulation [20]. Analysis of ChIP-seq and ATAC-seq using acetyltransferase *GCN5* mutants in Arabidopsis thaliana revealed that GCN5 regulates H3K14ac levels on target genes. The results suggest that GCN5 is involved in the biotic stress response through effects on the salicylic acid (SA) pathway [21]. However, the application of ATAC-seq to study changes due to drought has been relatively limited. In rice, OsNMCP1 positively responds to drought, and ATAC-seq analysis showed that *OsNMCP1* overexpression altered the chromatin accessibility of hundreds of genes related to drought tolerance and root growth. Additionally, overexpression of *OsSWI3C*, a subunit of the chromatin remodeling complex, led to a decrease in drought resistance. [22]. Therefore, the application of ATAC-seq is an important reference for revealing the molecular mechanisms under drought treatment.

Around the world, apple is one of the most popular fruits and an important economic crop with good nutrition. However, apples are always exposed to drought stress in some areas, which may limit apple yield and quality. There are many reports on the drought resistance of apples [23,24,25]. However, few studies of chromatin accessibility at the genome level have been reported in apples [26]. To investigate the effect of variation in chromatin accessibility on apples under drought treatment, we combined RNA-seq and ATAC-seq to explore potential cis-acting elemental loci and chromatin accessibility-related genes. The results of our study reveal chromatin changes that occur in apple drought, identify important transcription factors that act in the apple in response to drought stress, and can guide subsequent research on chromatin accessibility and the mechanisms of drought stress.

## 2. Results

### 2.1. Landscape of Accessible Chromatin Regions and Phenotype in Apple

**Figure 1:**
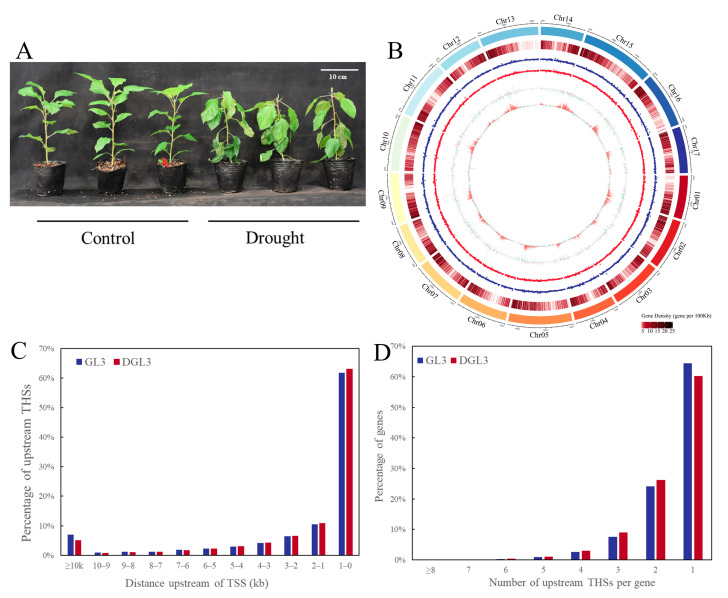
Plant phenotypes and characterization of ATAC-seq and RNA-seq data. (**A**) The phenotype of control and drought GL3. (**B**) Characteristics of ATAC-seq and RNA-seq, which are chromosome, gene density, distribution of GL3 ATAC data, distribution of DGL3 ATAC data, distribution of differential genes, and distribution of differential ATAC peaks from outside to inside. (**C**) The distribution of ATAC signals upstream of the gene TSS. (**D**) Number of ATAC signals upstream of genes.

One-month-old tissue culture plants of apple (GL3) seedlings with similar growth status were subjected to 12 days of drought treatment (DGL3). After the treatment, leaves clearly exhibited a water-deficient pendulous phenotype (Figure 1A). We used GL3 leaves without drought treatment or subjected them to 12 days of drought for subsequent ATAC-seq and RNA-seq analyses. For an overview of the data, we aligned the ATAC-seq versus RNA-seq data in Circos to compare drought and control treatments (Figure 1B). The results show a similar distribution of open chromatin in the drought and control groups, with only a few different regions. Differential open chromatin peaks clearly were more highly distributed in regions with higher gene density, and significantly more differential peaks were upregulated rather than downregulated after drought treatment. The distribution of differentially expressed genes was evenly spread relative to the open chromatin regions, with no significant differences in the number of up-and down-regulated genes (Figure 1B).

The preliminary analysis of the distribution of ATAC signal transposase hypersensitive sites (THSs) and the numbers of these sites upstream of a gene was similar to the numbers reported in other stress studies [17,19]. The analysis showed that ATAC signals were mostly distributed 0–1 kb upstream of the transcription start site (TSS) of genes. The signals gradually decreased with distance, but a small amount of open chromatin region remained more than 10 kb upstream of the TSS. There were slightly fewer THSs at 0–2 kb in the control group than the number in the drought treatment (Figure 1C). Most genes have only one THS upstream, and more than six THSs upstream of a gene were rare. The number of genes with only a single upstream THS was slightly decreased after drought treatment compared to pre-treatment, but the proportion of more than one THS was slightly increased (Figure 1D).

**Figure 2:**
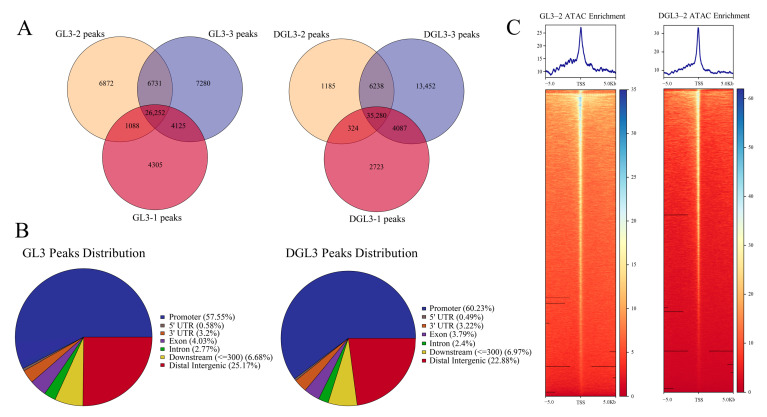
Characterization of ATAC signals after drought treatment of GL3 and DGL3. Different colors of the Venn plot represent different biological replications. (**A**) The Venn plots of three biological replicates of normal and drought treatment. (**B**) The distribution of the overlapping ATAC signals. (**C**) Distribution of partial samples of ATAC signals in the TSS region. Bin = 1.

Signals of open chromatin were obtained by alignment, duplication, and peaks called from the paired-end sequencing of six libraries prepared from three biological replicates of control and drought treatments. All six samples had a mapping rate of over 70% and biological replicates were highly correlated (Figure S1). As described above, the THSs indicate the open regions of chromatin enriched by ATAC-seq. To obtain signals with greater confidence, we selected the intersection of three biologically replicated peaks as a high-confidence signal of THSs for further study (Figure 2A). Similar to what was found for Arabidopsis and rice [17,22], over 50% of the THSs in apples were distributed within the 3.5 kb upstream of the promoter (promoter defined as the TSS-3500 to +100 bp region). About 25% of the mapped THSs are in intergenic regions, 6% in the downstream regions, and 6% in gene body regions (exons and introns). THSs were marginally increased in promoter regions after drought, but there was no significant difference compared with the control (Figure 2B).

To explore the gene functions related to chromosome opening under drought, we used GO analysis to perform functional enrichment of genes associated with THSs in control and drought treatment. The results showed enrichment of genes related to metabolism, development, and stress stimulation in both treatments, suggesting that most accessible chromatin corresponds to housekeeping genes that are actively transcribed for normal plant growth (Figure S2). We then checked the THSs in the 5 kb near TSSs by heatmaps and signal plots (Figure 2C and Figure S3). The results showed many THSs near the TSSs of genes, indicating greater chromatin accessibility near TSSs in control and drought treatments. The similar distribution of open chromatin in the two treatments suggests that the open chromatin regions are required for pathways necessary for normal plant growth and development. Therefore, further analysis was required to filter the data to identify THSs connected with drought treatment.

### 2.2. Enriched Motif Analysis and Functional Analysis of ATAC Peaks in Control and Drought Treatments

**Figure 3:**
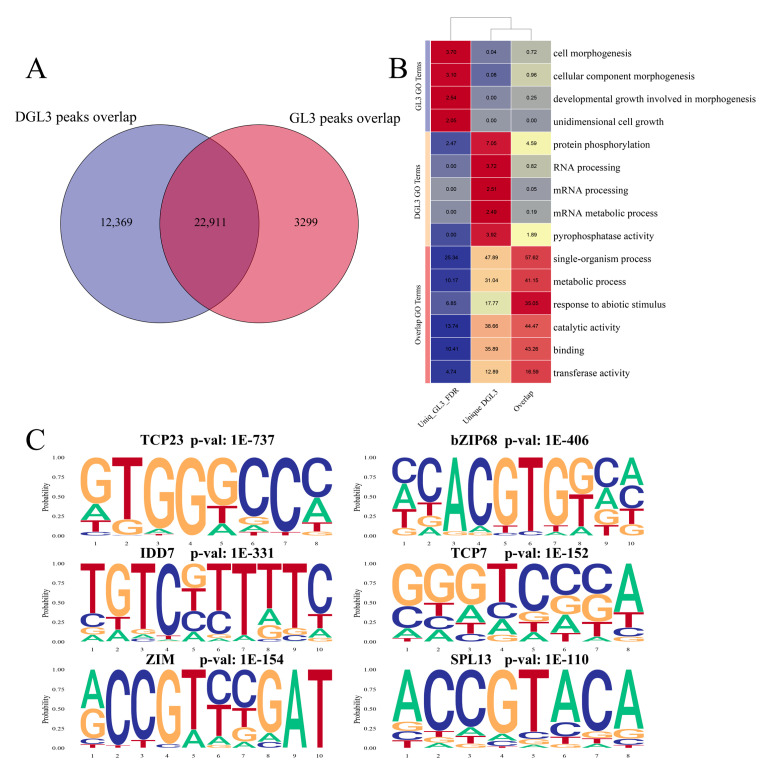
Functional enrichment of ATAC-seq results during the drought treatment. (**A**) The overlap of ATAC signals of the control and drought treatment. (**B**) The GO enrichment of ATAC signal-related genes of unique GL3, unique DGL3, and Overlap. Heatmap tagged with the number −log_10_ (FDR). Blue to yellow and then to red, representing a gradual increase in significance. (**C**) The motif analysis of overlap peaks in control and drought treatment.

To further screen the function of genes specifically linked to changes in areas of open chromatin with drought, we overlapped the THSs of the control and drought treatments. A total of 22,911 THSs were found in both the control treatment and in plants under drought, and these THSs likely indicate genes transcribed under normal growth conditions and required for plant development (Figure 3A). The analysis also revealed 12,369 and 3299 THSs specific to drought and control treatments, respectively, suggesting that these genes may have unique functions under different conditions. Functional analysis showed that genes associated with THSs specific to the control were mostly enriched in growth and development-related pathways. The genes associated with drought-specific THSs are mostly enriched in RNA pathways and protein phosphorylation (Figure 3B). The enrichment results suggest that chromatin opening under drought conditions can affect RNA processes and protein phosphorylation.

To further analyze the possible effects of the 12,369 THSs that specifically appear under drought treatment, we looked for genes associated with drought-related pathways. Analysis revealed that the 12,369 THSs were related to 10,457 genes. These 10,457 genes include 710 transcription factor genes, containing 65 *AP2/EREBP* family proteins, 91 *MYB* family proteins, 39 *NAC* family proteins, 21 *bZIP* family proteins, 50 *WRKY* family proteins, and other drought-related transcription factors. As protein phosphorylation is one of the common and critical pathways in signal transduction under drought treatment, we performed a further analysis of 309 genes related to protein phosphorylation in the enrichment result. Among these genes, 11 were Ca^2+^-dependent protein kinases (*CPKs/CDPKs*), which recognized Ca^2+^ signals to phosphorylate ABA effectors in guard cells. ABA can induce H_2_O_2_, which inhibits ABA-INSENSITIVE1 (ABI1) and ABI2, and thus alleviates the inhibition of GUARD CELL HYDROGEN PEROXIDE-RESISTANT1 (GHR1) by ABI2 [1]. *ABI2* was also found as a protein phosphorylation-related gene. The 309 genes related to protein phosphorylation included seven Calcineurin B-like protein-interacting protein kinases (*CIPKs*), five Mitogen-activated protein kinase kinase kinases (*MAPKKKs*), three Salt Overly Sensitive3 (*SOS3*)-interacting proteins and mitogen-activated protein kinases 3 (*MPK3*) with important roles in signal transduction [1,27,28]. The results suggest that drought-induced specific chromatin opening may play an important role in signal transduction under drought treatment.

Enrichment analysis of motifs for the overlap THSs identified additional transcription factors genes such as *TCP23*, *TCP7*, *bZIP68*, *IDD7*, *ZIM*, and *SPL13* (Figure 3C). Previous studies reported that TCP23 and TCP7 may be involved in flowering time control and plant development [29,30]. IDD7 plays a negative regulatory role in the growth and development of plants [31]. SPL13, bZIP68, and ZIM are involved in stress response and development in different plants [32,33,34,35]. These overlapped THSs-related transcription factors are clearly enriched in pathways involved in growth and development and may play additional roles during drought treatment.

### 2.3. Differential Analysis Reveals Dynamic Changes in Chromatin Accessibility and Transcriptome after Drought Treatment

**Figure 4:**
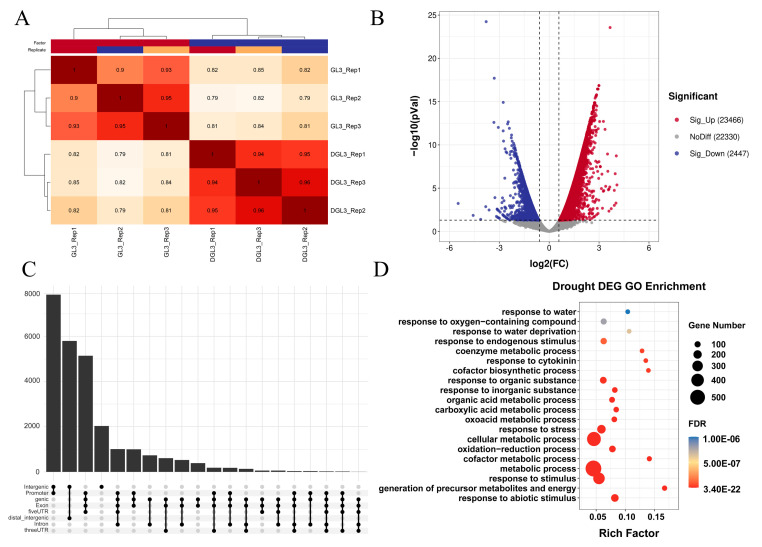
Results of differential ATAC analysis. (**A**) Correlation heatmap of six ATAC samples. Darker red means higher correlation. (**B**) Up- and down-regulation of differential ATAC signal. Genes marked in red are significantly up-regulated genes and those marked in blue are significantly down-regulated genes. (**C**) The distribution of differential ATAC signal intensity at different locations. (**D**) The GO enrichment of differentially expressed genes. Darker red means higher significance.

To better visualize the loci that showed changes in chromatin opening in response to drought, we next performed a differential analysis of the ATAC-seq data between control and drought treatments. There was a high correlation between the three biological replicates for each treatment (Figure 4A). A total of 25,913 differentially enriched ATAC-seq peaks representing differentially accessible regions (DARs) were identified with log2 (Fold Change) ≥ 0.6 and FDR < 0.05. The results showed that the number of DARs after drought (23,466 peaks) was significantly greater than the number of down-regulated DARs (2447 peaks) (Figure 4B and Table S1), a finding similar to the results of previous studies in rice [22]. The genomic distribution of DARs was similar to the distribution of THSs under single treatment conditions (Figure 2B and Figure S4A), with most signals in promoter and intergenic regions (Figure 4C). Finally, we examined the functional enrichment of genes adjacent to DARs and showed that both up-and down-regulated genes were significantly enriched in GO terms involving metabolism, stimulus, and binding. (Figure S4B,C). Although the enrichment results show a correlation with drought, many pathways are involved, suggesting that changes in chromatin accessibility could be a basal response that must be combined with other factors to have an effect.

To combine the chromatin accessibility data with gene expression data, we performed the differential analysis of the transcriptome. Heatmap and difference volcano plots analysis showed that the number of down-regulated genes (658) was slightly more than the number of up-regulated genes (585), and the three biological repeats were clearly clustered into one class (Figure S5A,B). The total number of DEGs (1243) was significantly lower than the number of changes in DARs (Figure 4B). DARs are predominately up-regulated genes (Figure 4B). However, the number of down-regulated DEGs is slightly more than up-regulated genes in transcriptome data, suggesting that the variation of DARs is more drastic than DEGs. For GO enrichment analysis, DEGs were significantly enriched in drought-related GO terms, including abiotic stresses, metabolic processes, and water response (Figure 4D), suggesting a high correlation of DEGs with drought. Compared to DEGs, DAR-related genes were generally assigned in most GO terms (Figure S4B,C). Transcriptome differential analysis under drought showed that DEGs were significantly different from DARs. This may suggest that changes in chromatin accessibility as a primary response may have different effects through various regulatory pathways. Combined analysis of RNA-seq and ATAC-seq can screen open chromatin regions with significant effects on gene expression.

### 2.4. Combined Analysis Revealed Chromatin Opening Dependent Pathways and Associated Genes

**Figure 5:**
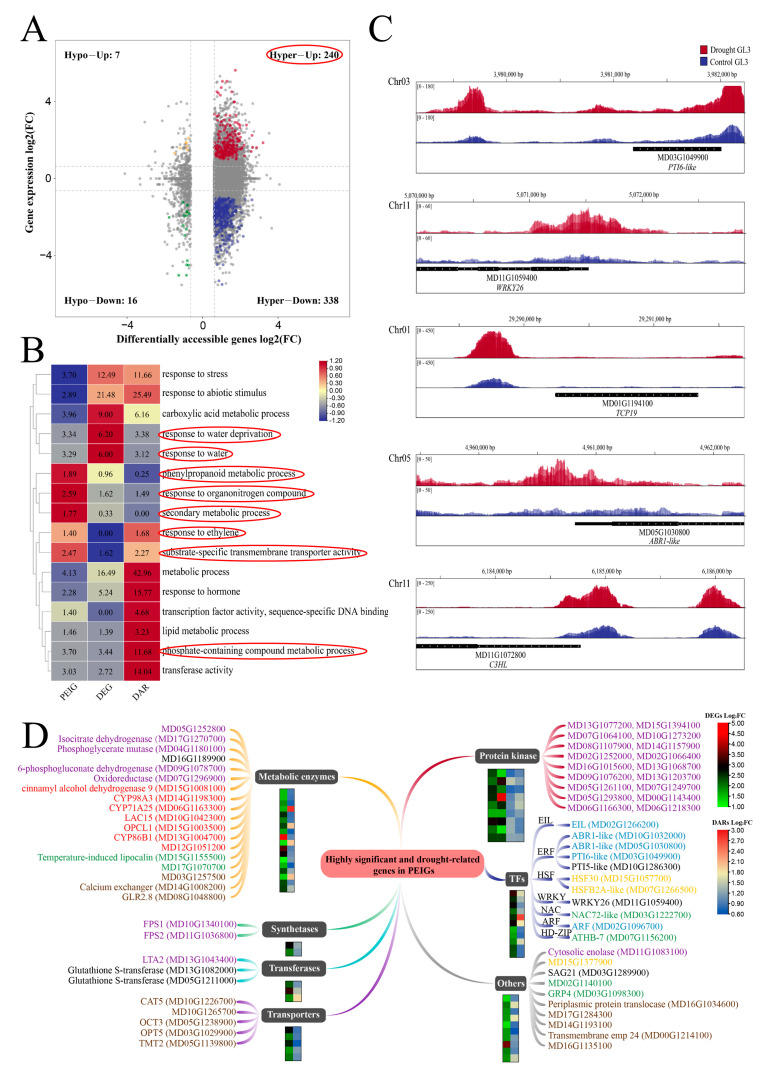
The association analysis of ATAC-seq and RNA-seq. (**A**) The pairwise comparisons of DEGs and differential ATAC-associated genes. The pathways marked with red boxes are used for further analysis in (**D**). (**B**) Functional enrichment of PEIGs compared with DEGs and DARs. Heatmap tagged with the number −log_10_ (FDR). (**C**) ATAC signaling distribution in the upstream promoter region of related transcription factors in PEIGs. (**D**) Annotation of significantly enriched pathway genes in PEIGs relative to DEGs and DARs, and water deprivation pathway related genes (marked by the red box in (**B**)). The involved pathways are marked with different colors: purple for phosphate-containing compound metabolic process, red for secondary metabolic process and phenylpropanoid metabolic process, yellow for response to organonitrogen compound, blue for response to ethylene, brown for substrate-specific transmembrane transporter activity, green for response to water deprivation, and black for multiple pathways.

The opening of chromatin near promoters may promote gene expression by facilitating the binding of RNA polymerase II, or by enhancing the binding of transcriptional activators or repressors. We identified genes with drought-induced changes in chromatin accessibility associated with the expression alterations, by overlapping the DAR-related genes and DEGs (Figure 5A and Figure S6A). Among them, a total of 240 genes were identified with both up-regulated chromatin accessibility and up-regulated expression levels (Figure 5A and Table S2), suggesting the products of these genes likely act in drought stress. Genes with greater promoter-accessibility and increased expression (PEIGs) were classified by gene type based on annotation (Figure S6B and Table S2). These PEIGs were also subjected to functional enrichment analysis with DEGs and DARs (Figure 5B). DARs were more significantly enriched in metabolism, hormones, transferase activity, and DNA binding, while DEGs were notably abundant in pathways such as water stress. As shown in Figure 5B, PEIGs were more prominently enriched in pathways related to phenylpropanoid metabolism, secondary metabolism, ethylene response, and organonitrogen compound response. Thus, PEIGs may play a more significant role in secondary metabolism and ethylene response compared to DARs and DEGs. We selected PTI6-like, WRKY26, TCP19, ABR1-like, and C3HL, as representative PEIGs and analyzed the distribution of THSs in their promoter regions (Figure 5C). WRKY26 and TCP19 are associated with abiotic stresses [36,37], and PTI6 and ABR1 may be involved in biotic and abiotic stresses [38,39]. These genes may be differentially expressed due to variations in chromatin accessibility indicating important roles in drought response in apples.

For further analysis of PEIGs, we selected significant enrichment and water stress-related PEIG genes for more detailed annotation (Figure 5D). As drought-specific THSs-related genes contain many phosphorylation-related kinases, we also provide detailed annotation of genes related to the phosphate-containing compound metabolic process. Most of the genes involved in metabolic enzymes are derived from phosphorus metabolism and secondary metabolic processes, including phenylpropanoid metabolism. These metabolism-related enzymes contain oxidoreduction and cytochrome related enzymes, and these metabolic enzymes may be regulated by chromatin accessibility under drought in apples. Among the selected genes, 18 protein kinases were included, possibly indicating that drought-related phosphorylation pathways may be influenced by chromatin accessibility. Among transcription factor genes, ERFs are ethylene-response factors, that act in stress response. Water deficiency-related transcription factor genes include NAC72-like, ATHB7, and metabolic enzymes, and these genes probably participate in the response to drought. There was enrichment of organonitrogen compound-related genes and HSFs, which were previously identified as acting in abiotic stress response [40]. Annotation results of water-deficient genes in PEIGs suggest that genes affected by open chromatin may participate in metabolic processes, transcription factor regulation, and protein phosphorylation.

We also overlapped the 12,369 THSs-related genes specific to drought and DEGs, to identify the genes with drought-specific expression changes with altered THSs. The results showed that a total of 326 differential genes were related to drought-specific open chromatin (Figure S5C), and heatmap showed similar numbers of up- and down-regulated genes (Figure S6C). Further annotation of 326 drought-specific chromatin accessibility-related genes revealed significant enrichment in the photosynthesis, electron transport chain, jasmonic acid metabolic process, and lignin metabolic process pathways (Table S3). These genes include LOX2, LOX3, AOC4, ST2A, and PKT3, which are related to jasmonic acid metabolism. CAD9, CYP98A3, LAC15, OMT1, and peroxidase superfamily proteins, are related to lignin metabolism. Jasmonic acid and lignin have important roles in abiotic stress processes [41,42]. The specific chromatin opening of these genes under drought may regulate their differential expression and potential functions in drought response.

### 2.5. Identification of TFs and Enriched DEG Motif Analysis in Response to Drought Stress

**Figure 6:**
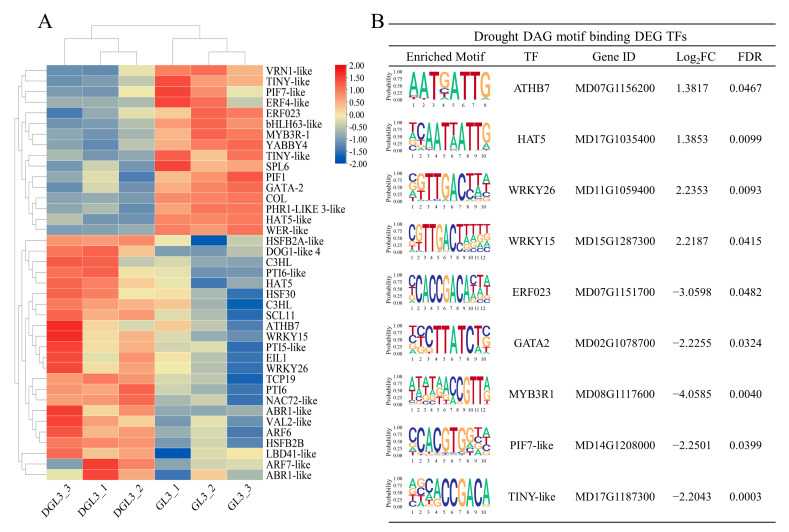
Differentially expressed transcription factors and differential ATAC signaling motif analysis. (**A**) Heatmap of the expression of differentially expressed transcription factors. (**B**) Differential ATAC signaling motif analysis enriched for differentially expressed transcription factors.

Chromatin opening regulates the binding of RNA polymerase II and transcription factors. The binding loci of transcription factors in DEGs were identified by using the motifs of DARs to search for potential transcription factors active under drought treatment. Differentially expressed transcription factors were identified in the DEGs, with 23 and 16 that exhibited transcription factors up- and down-regulated expression, respectively, after drought (Figure 6A). Combined with the motifs of the DARs, nine differentially expressed transcription factors with significant chromatin binding motifs under drought were finally obtained (Figure 6B). ATHB7, MYB3R1, and ERF023, were previously shown to respond to drought [43,44,45]. The family of WRKY transcription factors acts in plant response to stress [46,47]. PIF7 and TINY were identified in drought stress-related pathways [48,49]. DEGs with differential chromatin accessibility motifs may act in drought regulatory pathways.

**Figure 7:**
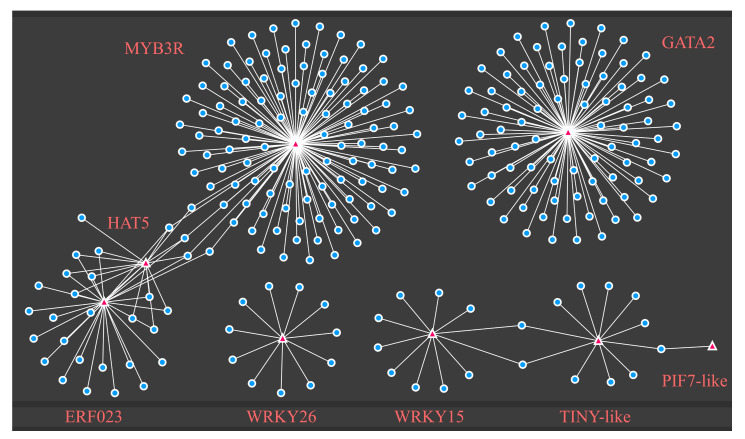
Visualization of the network of differentially expressed transcription factors with differential chromatin accessibility motifs. Co-expression network of differentially expressed genes connected with differential ATAC signaling motif enrichment transcription factors. Triangles represent differential ATAC signaling motif enrichment transcription factors and circles represent genes associated with these transcription factors.

To determine the possible regulatory functions of differential transcription factors with differential chromatin accessibility motifs, we performed a co-expression network analysis of the differentially expressed genes. Co-expression network analysis was applied by screening with *p* value < 0.01 and PCC > 0.9 (Figure 7). The analysis showed that all transcription factors genes except ATHB7 are associated with differentially expressed genes. Detailed functional analysis showed that TINY-like and WRKY15 related genes were more significantly enriched in functional terms of abiotic stress, external stimuli, and oxidative stress. GATA2, HAT5, and MYB3R1, related genes were obviously abundant in photosynthetic, energy, and metabolic pathways. Genes related to ERF023 were enriched in pathways involved in abscisic acid, catabolism, and genes linked to WRKY26 were associated with ethylene response and immune response (Table S4). The factors likely regulate these genes as part of drought response in apples.

### 2.6. Verification of Changes in Genes Expression in Response to Altered Chromatin Accessibility under Drought Treatment

**Figure 8:**
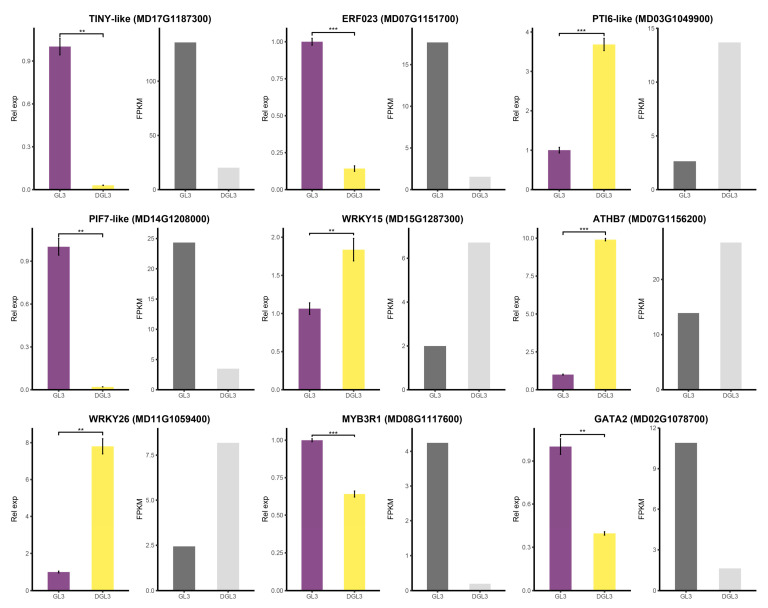
Relative expression of differentially expressed transcription factors measured by RT-qPCR and RNA-seq. The RT-qPCR and RNA-seq result of seven differential transcription factors with differential chromatin accessibility motifs and two PEIGs. The significance of comparisons was determined by Student’s *t*-test. Under each gene ID, the left figure shows RT-qPCR and the right figure shows RNA-seq results. ‘**’ at 0.01 level. ‘***’ at 0.01 level.

Alterations in chromatin accessibility may affect gene expression to impact plant drought resistance. To verify the reliability of the results above, we performed RT-qPCR validation of some of the differential transcription factors with chromatin accessibility changes (Table S5). TINY-like, ERF023, PTI6-like, PIF7-like, WRKY15, ATHB7, WRKY26, MYB3R1, and GATA2, were selected as genes exhibiting changes in chromatin accessibility under drought treatment. The RT-qPCR results are consistent with the trend of the transcriptomic data, further demonstrating the response of these transcription factors to drought (Figure 8). Role of ATP-dependent chromatin remodeling ATPases is a common chromatin remodeling mechanism in plants [50]. We identified an ATP-dependent helicase BRM (MD01G1206100) in the differential expressed genes of transcriptome data. We also selected additional chromatin remodeling factors to verify their responses under drought treatment. The RT-qPCR analysis showed that the changes in expression of six chromatin remodeling factors agreed with the transcriptome data. The differentially expressed chromatin remodeling factor BRM (MD01G1206100) was more significantly elevated than the six chromatin remodeling factors under drought (Figure S7), demonstrating its possible activation by chromatin accessibility changes after drought. The result showed no significant differences in chromatin remodeling factor LHP1 (MD07G1081800) and BRM-like (MD07G1134800) under drought and control treatment. PKL_X2 (MD08G1058800), PKL X1 (MD15G1037700), and BRM X1 (MD01G1065800), were down-regulated after drought (Figure S7), suggesting their possible involvement in the drought resistance process of apple. The effects of these four chromatin remodeling factors in drought should be verified by further experiments.

## 3. Discussion

With constantly changing external environments, plants must make changes in gene expression to survive. Drought is important abiotic stress in some areas and can inhibit plant growth and yield. Epigenetic effects in plant drought stress processes have been identified in many plant studies [51,52]. The relationship between DNA methylation and histone modification in drought stress is clear, but few studies have explored chromatin remodeling in response to drought. ATAC-seq is widely used in model plants as a simple way to study chromatin accessibility [17,53], with high specificity [54]. In this study, we combined RNA-seq with ATAC-seq to identify transcription factors important to drought stress and the genes they regulate.

The distribution of THSs changed between drought and control conditions, with greater enrichment in the 0–1 kb range in the treatment group compared to the drought treatment, with most promoter regions containing only one THS similar to previous results [17,19]. The distribution of THSs in apples is closer to that reported for Arabidopsis and rice, but slightly different from the wider distribution of THSs for plants with larger genomes. Genome size may not be strictly correlated with the distribution of THSs, as the distribution of THSs in M. truncatula (~480 Mb), which is similar in size to the rice genome (~400 Mb), has a more similar pattern of THSs to that of tomato (~820 Mb), although the genome size of apple (~700 Mb) is similar with that of tomato [17]. The THSs showed no significant differences in the TSS region in the treatment compared with the control, and there were no significant differences in the functional enrichment results. This may be because most signaling related to THSs is associated with plant growth and development. Differential analysis in conjunction with transcriptome analysis may be required to determine which areas of chromatin opening are directly associated with drought. Overlapping the THSs in the drought and control treatments revealed common chromatin open motif enrichment of TCP23, bZIP68, IDD7, TCP7, ZIM, and SPL13. These transcription factors are all involved in plant developmental processes [29,30,31,32,33,34,35], suggesting that the overlapping THSs act in processes related to plant growth or development. Simultaneous enrichment analysis of THSs-related genes specific to drought treatment showed enrichment of drought-specific genes related to RNA process protein phosphorylation and enrichment of control-specific genes related to development. Thus, drought alters many THSs loci that are developmentally relevant under non-drought conditions and loci with drought-specific THSs may have an impact on protein phosphorylation and RNA processes.

Differential analysis of the ATAC-seq results showed a total of 25,913 DARs, similar to the 24,841 DARs identified in rice drought [22]. The number of increasing DARs was much higher than the number of decreasing ones, with the same trend of DARs found in both cold and drought conditions [19,22]. Chromatin may generally be more open under stress. The analysis of DEGs in the RNA-seq results showed fewer up-regulated genes than the number of down-regulated genes, which was similar to the finding of a previous study [55]. ATAC-seq and RNA-seq differential analysis combined with the previous enrichment analysis of single treatments suggest that changes in THSs are highly related to plant response to stress. The high number of DARs may indicate these regions contain genes with multiple epigenetic modifications and play different roles in different pathways. Previously, it was demonstrated that changes in THSs did not correlate significantly with changes in gene expression [17]. DARs may be associated with DNA methylation, histone modification, or non-coding RNA modification and regulation under specific conditions. DARs may reflect fundamental changes that occur as part of the plant response to drought, and improved histological data may be needed to more deeply analyze the effect of chromatin opening on plant drought resistance.

The regulation of transcription factors correlates with the degree of chromatin opening, so we looked for differentially expressed transcription factors and motifs that were enriched in DARs. Nine transcription factor genes, *ATHB7*, *HAT5*, *WRKY26*, *WRKY15*, *ERF023*, *GATA2*, *MYB3R1*, *PIF7-like*, and *TINY-like,* were identified. These transcription factor genes may be affected by changes in the degree of chromatin opening and thus play some role in the apple drought process. Among them, *ATHB7*, *MYB3R1*, and *ERF023,* were previously reported in drought pathways [43,44,45]. *WRKY* transcription factors act during stress [46,47]. *PIF7* and *TINY* are involved in drought-related pathways [48,49]. *GATA2* was linked to brassinolide and auxin [56,57], and *HAT5* was implicated in hypocotyl elongation in Arabidopsis [58]. Further enrichment results of co-expression network analysis showed that these transcription factors may influence drought resistance through gene regulation in different pathways. Determining the specific relationships between these nine transcription factors and regulated genes in drought resistance will be an important goal of future research.

The co-analysis of RNA-seq and ATAC-seq identified 240 genes with up-regulated chromatin accessibility and up-regulated expression, all 240 genes having at least one THSs in promoter regions. Previous ATAC studies demonstrated that genes that are simultaneously upregulated are more likely to play important functions under drought [22]. These genes were more significantly enriched in pathways related to phenylpropanoid metabolism, secondary metabolism, ethylene response, and organonitrogen compound response. We analyzed the distribution of the promoter region peaks for *PTI6-like*, *WRKY26*, *TCP19*, *ABR1-like,* and *C3HL*, as these transcription factor genes were previously shown to be involved in stress-related pathways in previous studies [36,37,38,39,59]. Increased chromatin accessibility in promoter regions may allow the binding of RNA polymerase II and other regulatory factors, which can promote transcription and gene expression to respond to drought. However, 16 genes with down-regulated chromatin accessibility and expression were also identified in our study. These gene regions may appear to have chromatin tightness after drought, but we are not clear about the relationship between chromatin tightness and expression in this case. It may be influenced by a variety of factors, including the effects of reduced RNA polymerase II, histone modifications, etc. Therefore, further studies are needed to explain the reduction in expression caused by the lower chromatin accessibility after drought.

Chromatin remodeling can regulate the accessibility of genomic DNA and consequently promote or obstruct transcription. Among the ATP-dependent chromatin remodeling factors, SWI/SNF and CHD have been reported in water stress [3]. Among these drought-responsive chromatin remodeling factors, the SWI/SNF subfamily contains BRAHMA (BRM), SPLAYED (SYD), and MINUSCULE (MINU). In Arabidopsis, the BRM complex has been associated with ABA and drought response, and BRM mutants showed ABA hypersensitive response and enhanced growth arrest [10]. The CHD subgroup chromatin remodeler PKL was also associated with ABA response [15]. To explore the drastic changes in chromatin accessibility under drought, we selected six related chromatin remodeling factors, including BRM and CHD, for expression validation based on changes in FPKM. The results showed that the DEG of *BRM* (MD01G1206100) showed significant differences at 0.01 level, while *PKL X2* (MD08G1058800), *PKL X1* (MD15G1037700), and *BRM X1* (MD01G1065800), showed significant differences at the 0.05 level. These genes probably contribute to the dramatic changes in chromatin accessibility during drought, which may in turn affect drought resistance in apples.

## 4. Material and Methods

### 4.1. Plant Materials and Drought Treatment

After tissue culturing, one-month transplants of “GL3” (*Malus* × *domestica*) were used for drought treatment. “Royal Gala” (*Malus* × *domestica*) progeny GL3 was widely used for genetic transformation due to its high regeneration rate. The study of the chromatin accessibility of GL3 after drought will be beneficial for later transgenic studies.

To study the relationship between chromatin accessibility and transcriptome of GL3 in control and drought treatment. GL3 were grown after 1 month of subculture, 1.5 months of root culture, and one-month transplants were cultivated in plant culture rooms (temperature 25 °C, humidity 50%) in water-saturated and constant weight soil for 12 days, while controls were watered normally. The above-ground parts of Gl3 were about 17 cm and the stem thickness were about 0.32 cm. After the water loss phenotype was obvious in the leaves, the whole live plant material was subjected to ATAC-seq and RNA-seq sequencing. RNA-seq and ATAC-seq use the same samples for sequencing, and RNA-seq is performed simultaneously with ATAC-seq. GL3 plants in the same state as the sequenced plants were also selected for subsequent RNA extraction and RT-qPCR analysis. The top 4th–6th leaves of every 3 GL3 plants were mixed as a biological replicate, followed by rapid storage of the 6 samples in liquid nitrogen before storage at −80 °C.

### 4.2. Library Construction and Analysis Process for RNA-seq Samples

Six samples for following transcriptome analysis. The total RNA was assessed using the NanoPhotometer^®^ spectrophotometer (IMPLEN, San Diego, CA, USA) to purify, Qubit^®^ RNA Assay Kit in Qubit^®^ 2.0 Flurometer (Life Technologies, Carlsbad, CA, USA) to concentrate and RNA Nano 6000 Assay Kit of the Bioanalyzer 2100 system (Agilent Technologies, California, USA) to integrate.

A total 20 ng RNA per sample was used for the RNA sample preparations. Library preparation for sequencing. Firstly, using Epicentre Ribo-zero™ rRNA Removal Kit (Epicentre, USA) to remove ribosomal RNA and cleaned up by ethanol precipitation. Subsequently, using rRNA-depleted RNA by NEBNext^®^ Ultra™ Directional RNA Library Prep Kit for Illumina^®^ (Nebraska, USA) to generate sequencing libraries.

cBot Cluster Generation System using TruSeq PE Cluster Kit v3-cBot-HS (Illumia, San Diego, California, USA) was used to perform index-coded samples cluster. On an Illumina Hiseq 4000 platform, the libraries were sequenced and 150 bp paired-end reads were produced.

The process of RNA-seq involves quality control, sequence alignment, and differential analysis. By deleting adapter-containing reads, ploy-N-containing reads, and low-quality reads from raw data, clean data was obtained. Clean data were used for quality control by FastQC v0.11.9 (http://www.bioinformatics.babraham.ac.uk/projects/fastqc/, accessed on 23 December 2021) with default parameters. After quality control, the clean reads were mapped to the reference genome [60] by HISAT2 v2.1.0 [60,61]. After the alignment, use samtools v1.9 with parameter -bq 1 [62] to get the sorted binary bam file after transformation and sorting. mRNA reads counting of sequenced Bam files combined with genome annotation files using Htseq v0.13.5 [63]. FPKM stands for fragments per kilobase of exon model per million mapped fragments (Table S6), and it is calculated using the following formula: FPKM = read counts/(mapped reads (Millions) × exon length (KB). To get significant differences, differential expression analysis of 6 samples expression matrix was performed using the R/DESeq2 [64]. The significance level was determined by using the corrected *p*-value (padj). The absolute value of log2(fold change) >1 with padj < 0.05 was defined as the different expression genes.

### 4.3. Library Construction and Analysis Process for ATAC-seq

ATAC-seq was performed as previously reported [65]. In a nutshell, nuclei were removed from samples and resuspended in the Tn5 transposase reaction mix. For 30 min, the transposition process was incubated at 37 °C. After transposition, equimolar Adapter 1 and Adapter 2 were introduced, and the library was amplified using PCR. Libraries were purified using AMPure beads after the PCR process, and library quality was evaluated with Qubit.

The index-coded samples were clustered using the TruSeq PE Cluster Kit v3-cBot-HS (Illumina, San Diego, CA, USA) on a cBot Cluster Generation System according to the manufacturer’s instructions. The library preparations were sequenced on an Illumina Hiseq platform after cluster creation, yielding 150 bp paired-end reads.

Adaptor sequences from Nextera were first removed from the reads using skewer v0.2.2 [66]. Bowtie2 v 2.3.5.1 [61] was used to align these reads to reference genome using default parameters. After transformation and sorting by samtools v1.9, PCR duplicates are removed using the sambamba v0.8.1 [67] with default parameters. Peak calling was performed with macs2 v2.2.7.1 [68] using ‘macs2 callpeak -q 0.05 -f BAMPE -g 625672265 --nomodel --extsize 70 --keep-dup all -B’. The reads density of the three replicates inside the region of peaks between various experiments was assessed and compared using R/Diffbind v3.3 [69] based on the edgeR technique for differential peak analysis. R/Chipseeker [70] was used to annotate the genomic characteristics attributed to the peaks, with a promoter defined as the 3500 to +100 bp area in proximity to the transcriptional start point (TSS).

To obtain high confidence peaks, intervene v0.6.5 [71] with default parameters was used for all peak overlapped and Venn plots. All motif analyses were performed using HOMER’s FindMotifGenome.pl v 4.11 [62] tool with the parameters of ‘-len 4,6,8,10,12’. Enrichment of reads in the TSS region using deeptools v3.5.1 with the parameters of ‘--referencePoint TSS -bs 1 -a 5000 -b 5000′. The quality of data in the manuscript is in the Table S7.

### 4.4. Differential Co-Expression Network Analysis and GO Enrichment

Differential transcription factors were obtained from the results of transcriptome differential analysis. In combination with ATAC-seq differential motif analysis, nine transcription factors with differential motif binding loci and differential expression were identified. The heatmaps of differential genes were made by TBtools [72]. To show the co-expression network of all the different genes were selected as candidates to calculate Pearson correlation coefficient (PCC) by using the cor.test in the R base packages. The connection of nine transcription factors which PCC > 0.9 and pVal < 0.01 to construct co-expression networks (Table S8). In Cytoscape [73], the nine transcription factors and the connections of different expressed genes were utilized to create basic co-expression networks. Enrichment analysis of differentially expressed genes or ATAC-seq results by Gene Ontology (GO) is implemented by agriGO v2.0. Differentially expressed genes were identified highly enriched by GO keywords with corrected *p*-values less than 0.05. The heatmaps of GO terms were made by TBtools.

### 4.5. RNA Extraction and RT-qPCR Analysis

RNAprep Pure Plant Kit (Polysaccharides&Ployphenolics-rich, DP441) was used to extract RNA from four different stages of fruit (Tiangen Biotech, Beijing, China). Primer-BLAST (https://www.ncbi.nlm.nih.gov/tools/primer-blast/, accessed on 22 March 2022) was used to create the primers utilized in the experiment (Table S5). HiScript^®^ II 1st Strand cDNA Synthesis Kit (+gDNA wiper) was used to make the first-strand cDNA (Vazyme, Nanjing, China). The internal reference gene is malate dehydrogenase (MDH) [74]. On a Bio-Rad CFX96TM system, real-time quantitative PCR (RT-qPCR) analysis was performed using ChamQ SYBR qPCR Master Mix (Vazyme, Nanjing, China).

## 5. Conclusions

Our study identified regions with differential chromatin accessibility after drought by using ATAC-seq in apples under drought treatment. The results revealed 23,466 peaks of upregulated chromatin accessibility and 2447 peaks of downregulated accessibility after drought. Combining the ATAC-Seq and RNA-seq data, 240 genes exhibited increased promoter-accessibility and expression increased genes (PEIGs) that may respond to drought were identified. Nine differentially expressed transcription factors with DARs binding loci were used to construct connected DEGs co-expression networks. Our results provide a theoretical basis for subsequent experiments on the changes in chromatin accessibility and gene expression during drought tolerance in apples.

## Data Availability

Publicly available datasets were analyzed in this study. This data can be found in the NCBI BioProject (BioProject, https://www.ncbi.nlm.nih.gov/bioproject/) under accession number PRJNA821644.

## Supplementary Materials

The following supporting information can be downloaded at: https://www.mdpi.com/article/10.3390/ijms231911191/s1.
