# Supplementary material for: Integrating ATAC-seq and RNA-seq Reveals the Dynamics of Chromatin Accessibility and Gene Expression in Apple Response to Drought
Source: Int J Mol Sci. 2022 Sep 23;23(19):11191. doi: 10.3390/ijms231911191 (PMC9570298; doi:10.3390/ijms231911191)
Supplement: Supplementary file 1 [file ijms-23-11191-s001.zip › ijms-1912352-supplementary materials caption.pdf]

Figure S1: The Spearman Correlation of all six samples; Figure S2: Distribution of other four samples' ATAC signals in the TSS region. Bin = 1; Figure S3: The enrichment of genes related to down-regulated DARs; Figure S4: The RT-qPCR results of selected ATP-dependent chromatin remodeling ATPases; Figure S5: The supplementary result of RNA-seq and the overlap of DEGs and drought-specific THSs; Figure S6: The expression pattern of the overlap of DEGs and DARs, PEIGs, and the overlap of drought-specific THSs and DEGs; Figure S7: The RT-qPCR results of selected ATP-dependent chromatin remodeling ATPase; Table S1: DGL3_vs_GL3_DARs; Table S2: Four-quadrant plot related genes and PEIGs; Table S3: GO enrichment of the overlap in drought-specific THSs and DEGs; Table S4: GO enrichment of transcription related DEGs in co-expression network; Table S5: Primers and RT-qPCR result; Table S6: FPKM of mRNAs; Table S7: The quality of data in the manuscript; Table S8: The connection in the co-expression network of DEGs.
