# Supplementary material for: Integrating ATAC-seq and RNA-seq Reveals the Dynamics of Chromatin Accessibility and Gene Expression in Apple Response to Drought
Source: Int J Mol Sci. 2022 Sep 23;23(19):11191. doi: 10.3390/ijms231911191 (PMC9570298; doi:10.3390/ijms231911191)
Supplement: Supplementary file 1 [file ijms-23-11191-s001.zip › Supplyment_Figture.pdf]

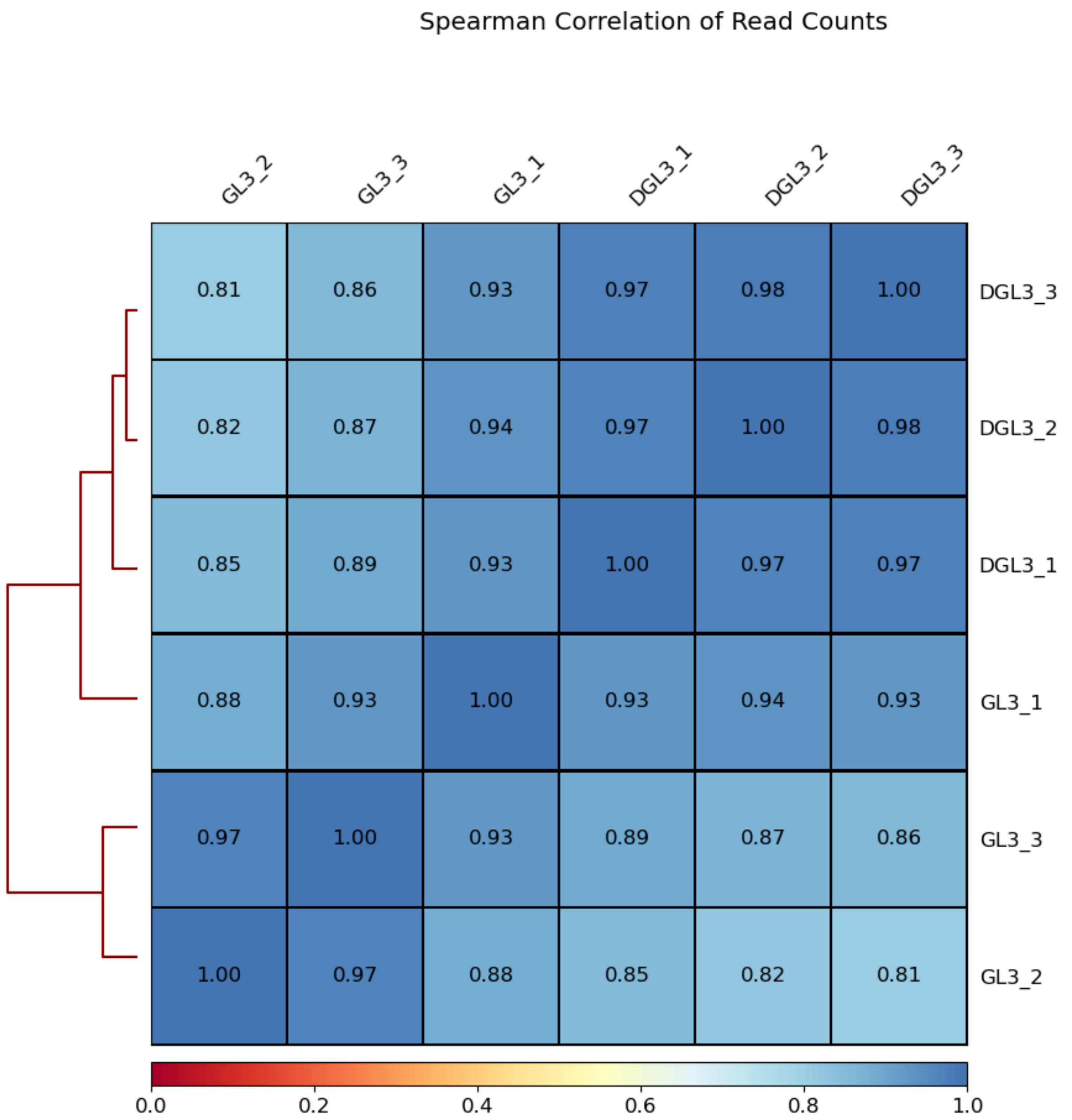


Figure S1. The Spearman Correlation of all six samples

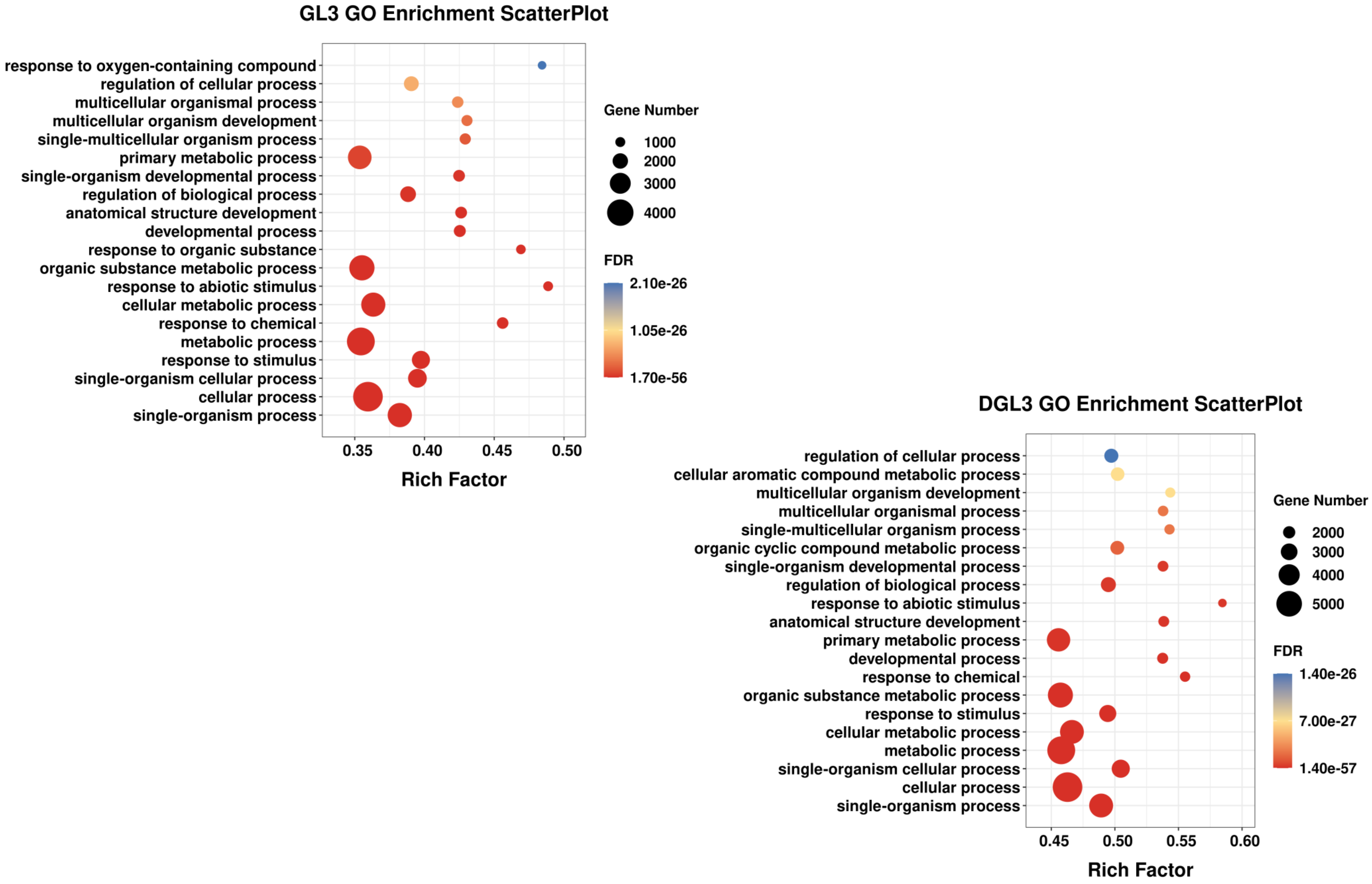


Figure S2. Functional enrichment of the overlap ATAC signals related genes.

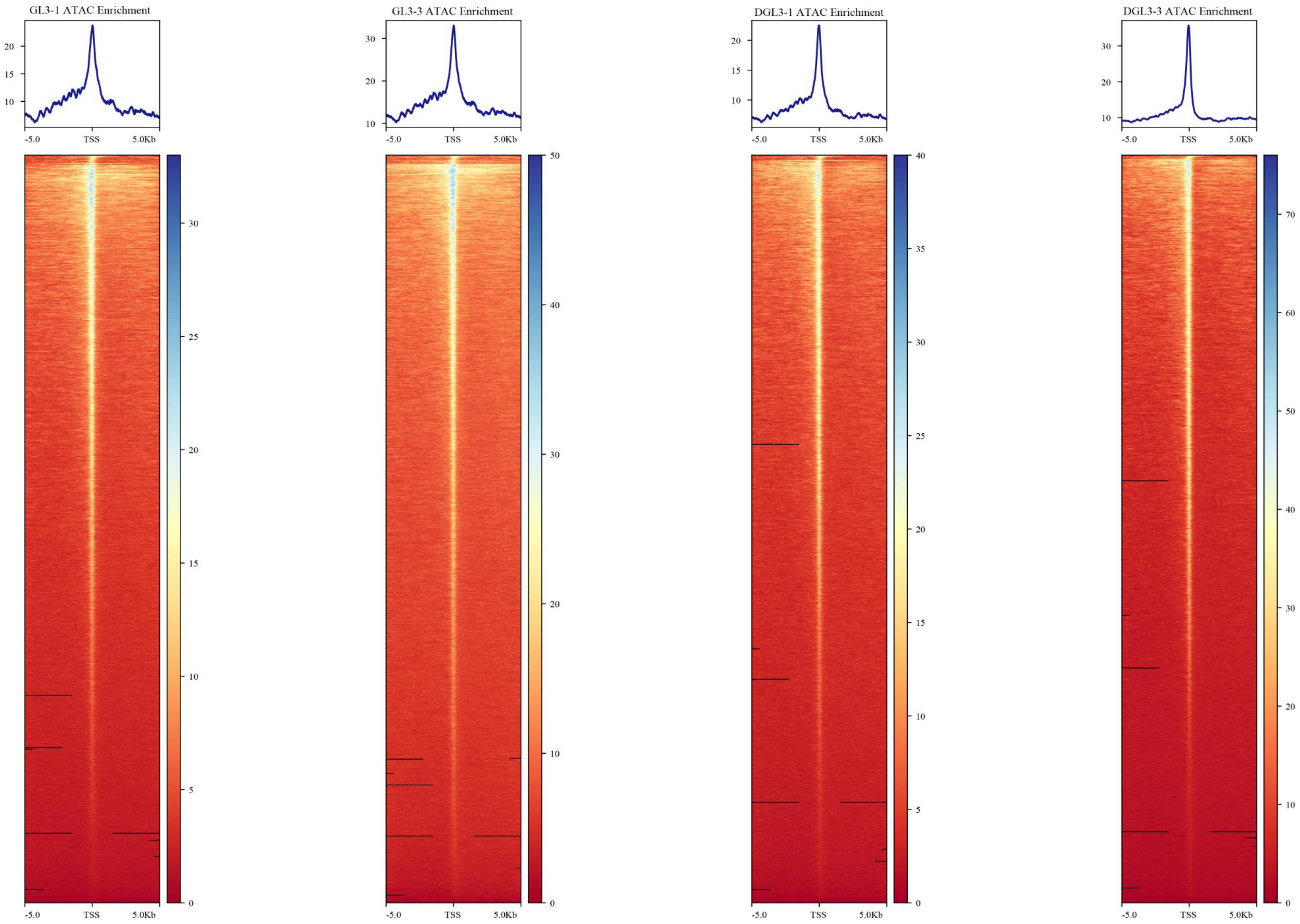


Figure S3. Distribution of other four samples' ATAC signals in the TSS region. Bin=1.

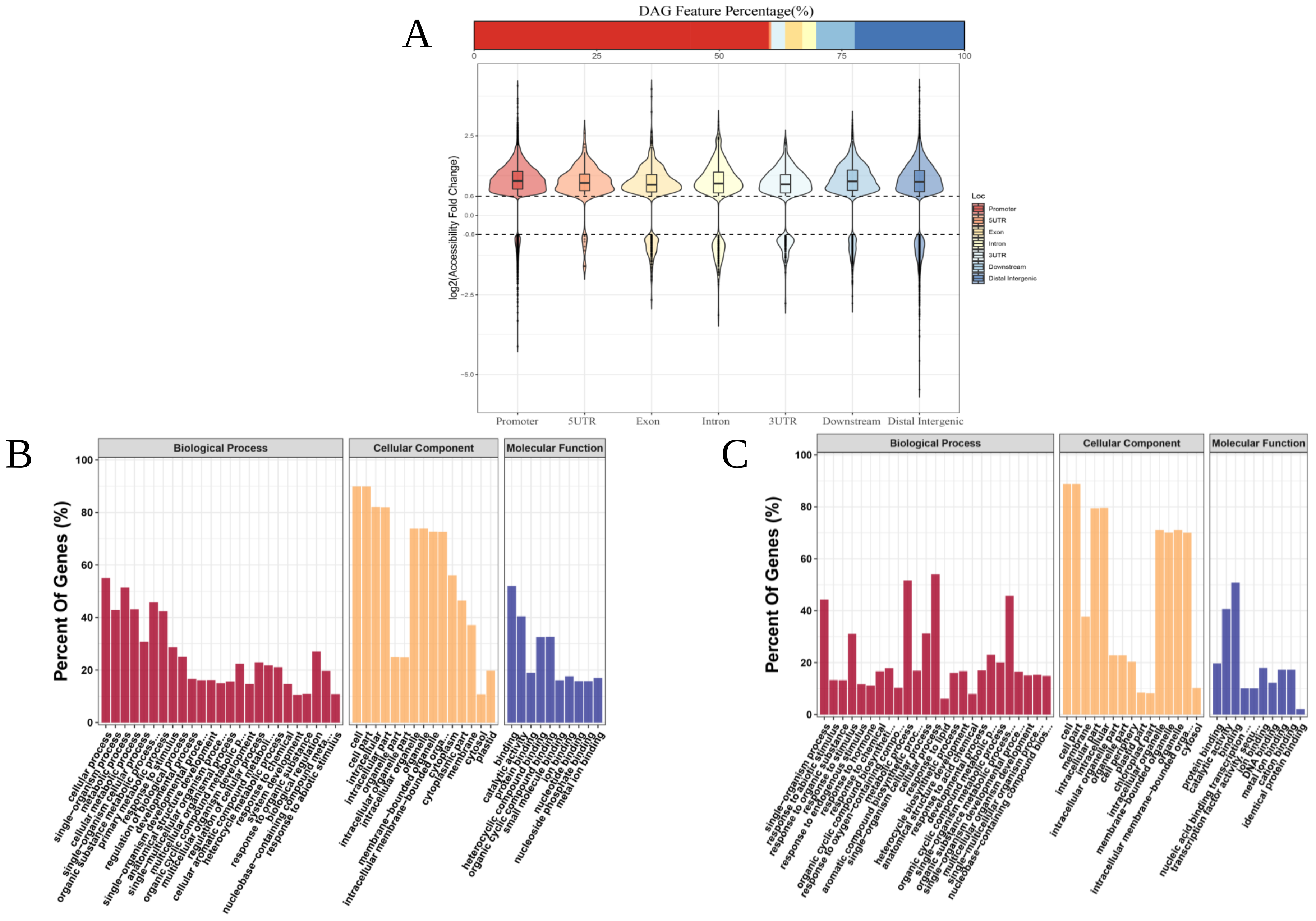


Figure S4. (A) The distribution of differential ATAC signal intensity at different locations. (B) The enrichment of genes related to up and (C) down-regulated DARs (Right figure).

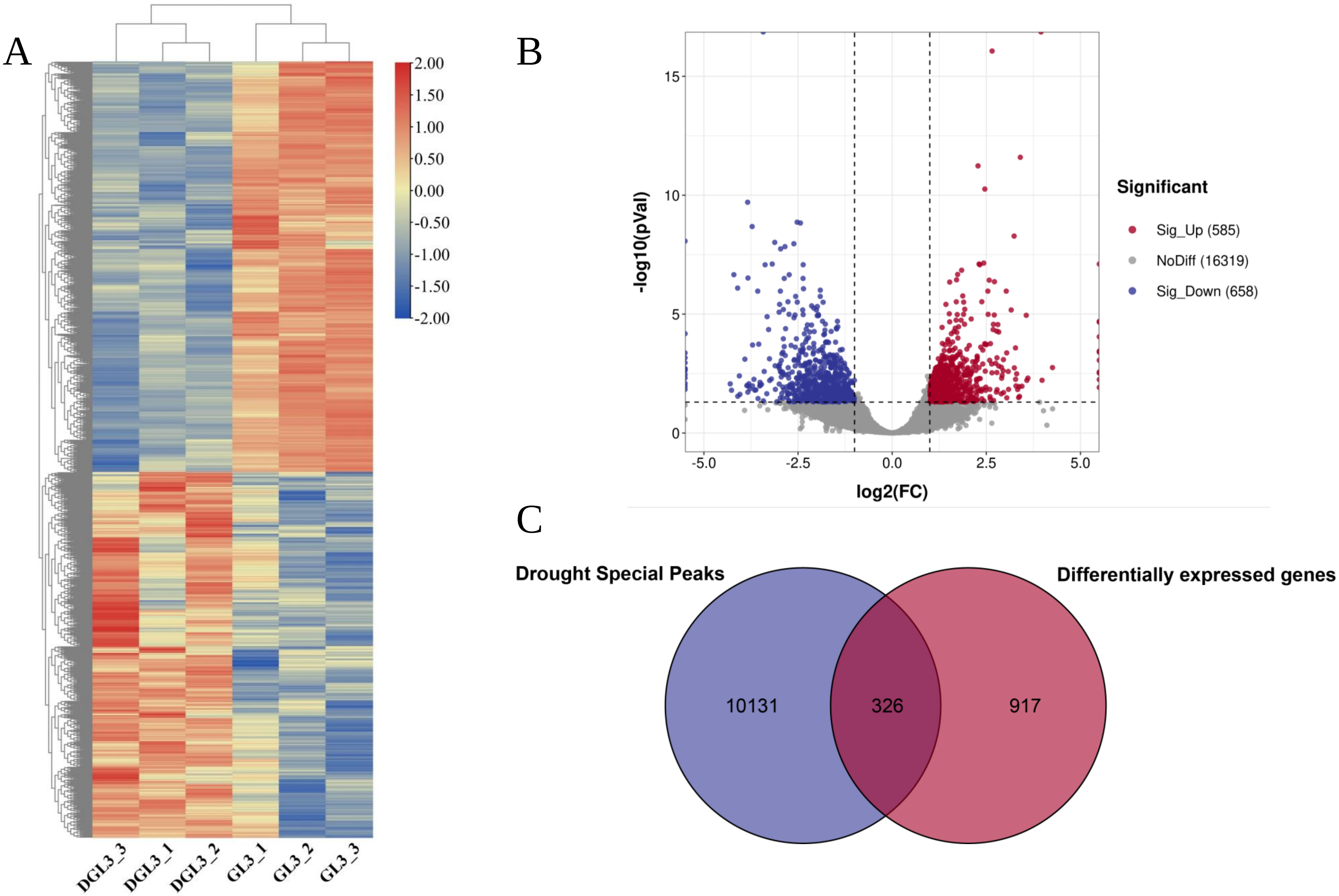


Figure S5. (A) The expression pattern of different expressed genes. (B) The volcano plot of the different expressed genes. (C) The Venn plots of drought-specific THSs and DEGs.

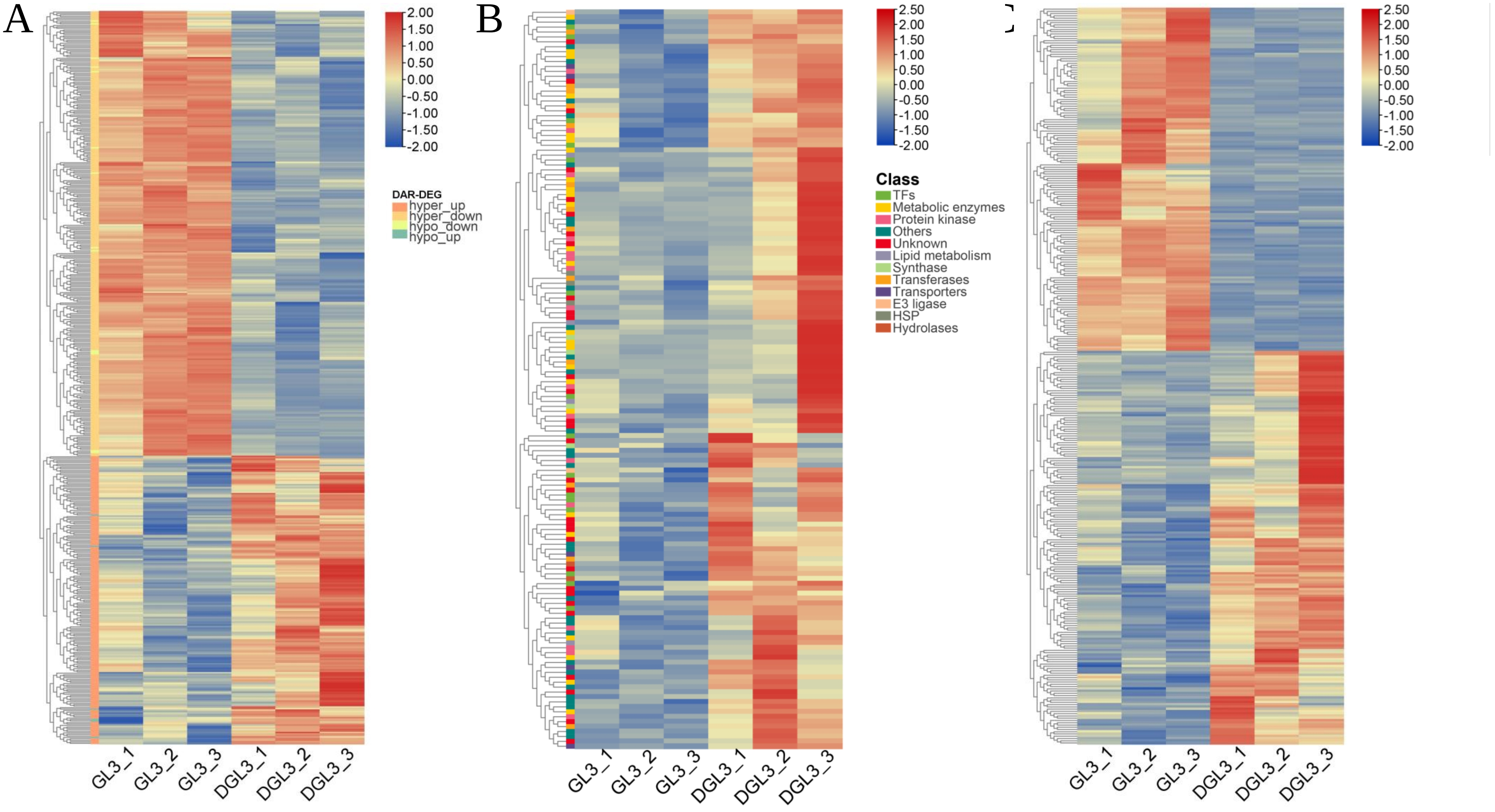


Figure S6. (A) The expression pattern of the overlap of DEGs and DARs. (B) The expression pattern of PEIGs. (C) The expression pattern of the overlap 326 genes in drought-specific THSs and DEGs.

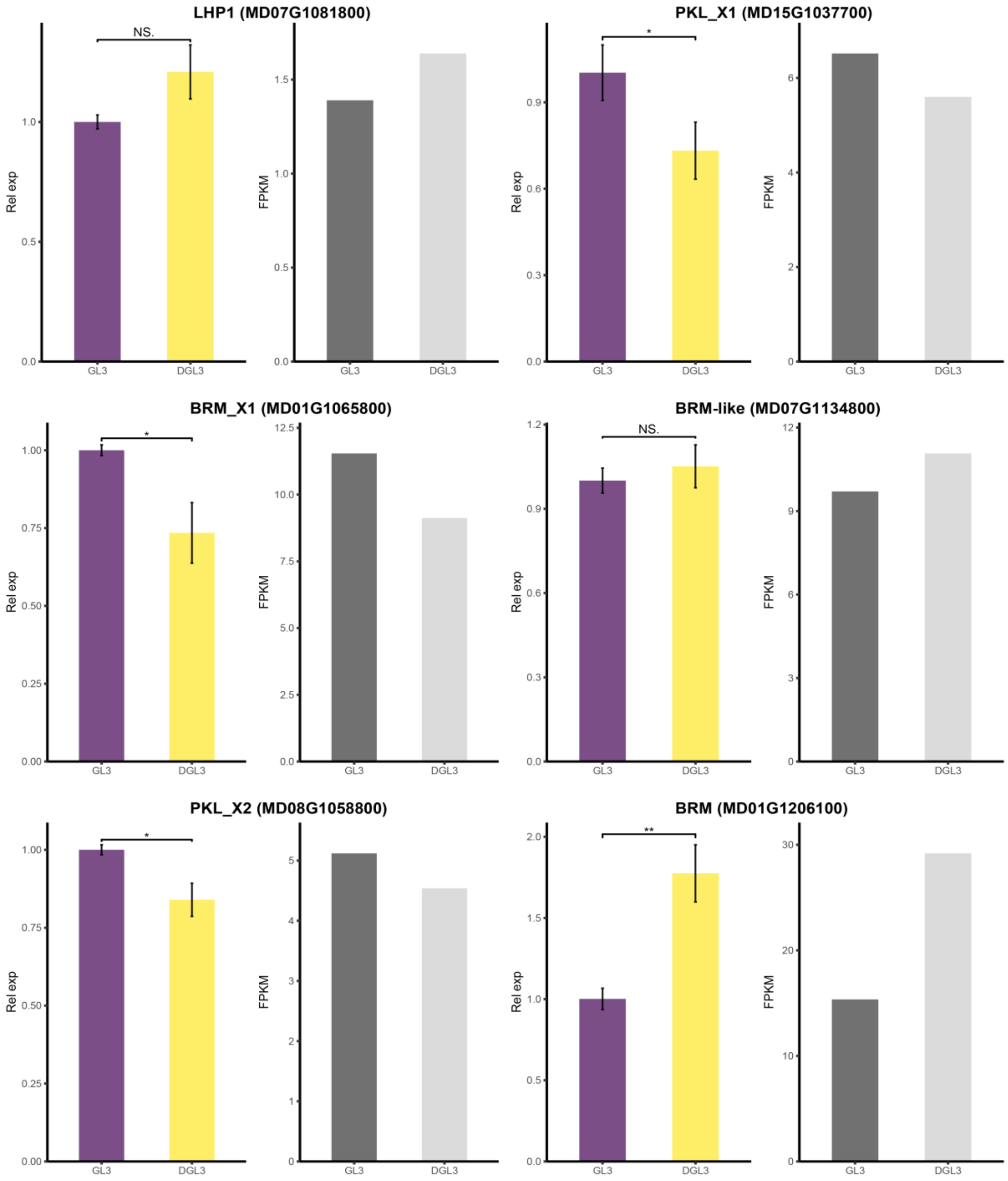


Figure S7. The RT-qPCR results of selected ATP-dependent chromatin remodeling ATPases.
